# Measuring Molecular Complexity

**DOI:** 10.1021/acscentsci.4c00697

**Published:** 2024-05-08

**Authors:** Louie Slocombe, Sara Imari Walker

**Affiliations:** † Beyond Center for Fundamental Concepts in Science, Arizona State University, Tempe, Arizona 85287-0506, United States; ‡ School of Earth and Space Exploration, Arizona State University, Tempe, Arizona 85287-1004, United States; § Santa Fe Institute, Santa Fe, New Mexico 87501, United States

In a scientific era focused on big data, it is easy to lose sight of the critical role of metrology—the science of measurement—in advancing fundamental science. However, most major scientific advances have been driven by progress in what we measure and how we measure it. An example is the invention of temperature,^1^ where before it, we could say one thing was hotter than another but without a standardized, empirical measure we could not say how much hotter. This is not unlike the current state in discussing complexity in chemistry,^2,3^ where we can say molecules are complex but lack an empirically validated standardization to confirm that one is more complex than another. In this issue of *ACS Central Science*,^4^ a set of experiments by Leroy Cronin and co-workers conducted at the University of Glasgow aim to change this by providing a new kind of measurement with a well-defined scale, a significant step toward a metrology of complexity in chemistry. Although the concept of quantifying molecular complexity is not new itself,^3^ the team leveraged principles from the recently developed theory of molecular assembly (MA) and related ideas^5^ to define a rigorous concept of a scale for complexity, connected to a theory for how evolution builds complex molecules.^6,7^ They show how the complexity of molecules on this scale can be inferred from standard laboratory spectroscopic techniques, including nuclear magnetic resonance (NMR), infrared (IR) spectroscopy, and tandem mass spectrometry (MS/MS). The robust validation of the inferred complexity across a multimodal suite of techniques instills confidence in the objectivity of the complexity scale proposed and the reliability of its resultant measurement.

## What is molecular assembly theory, and why is it useful?

**Figure 1:**
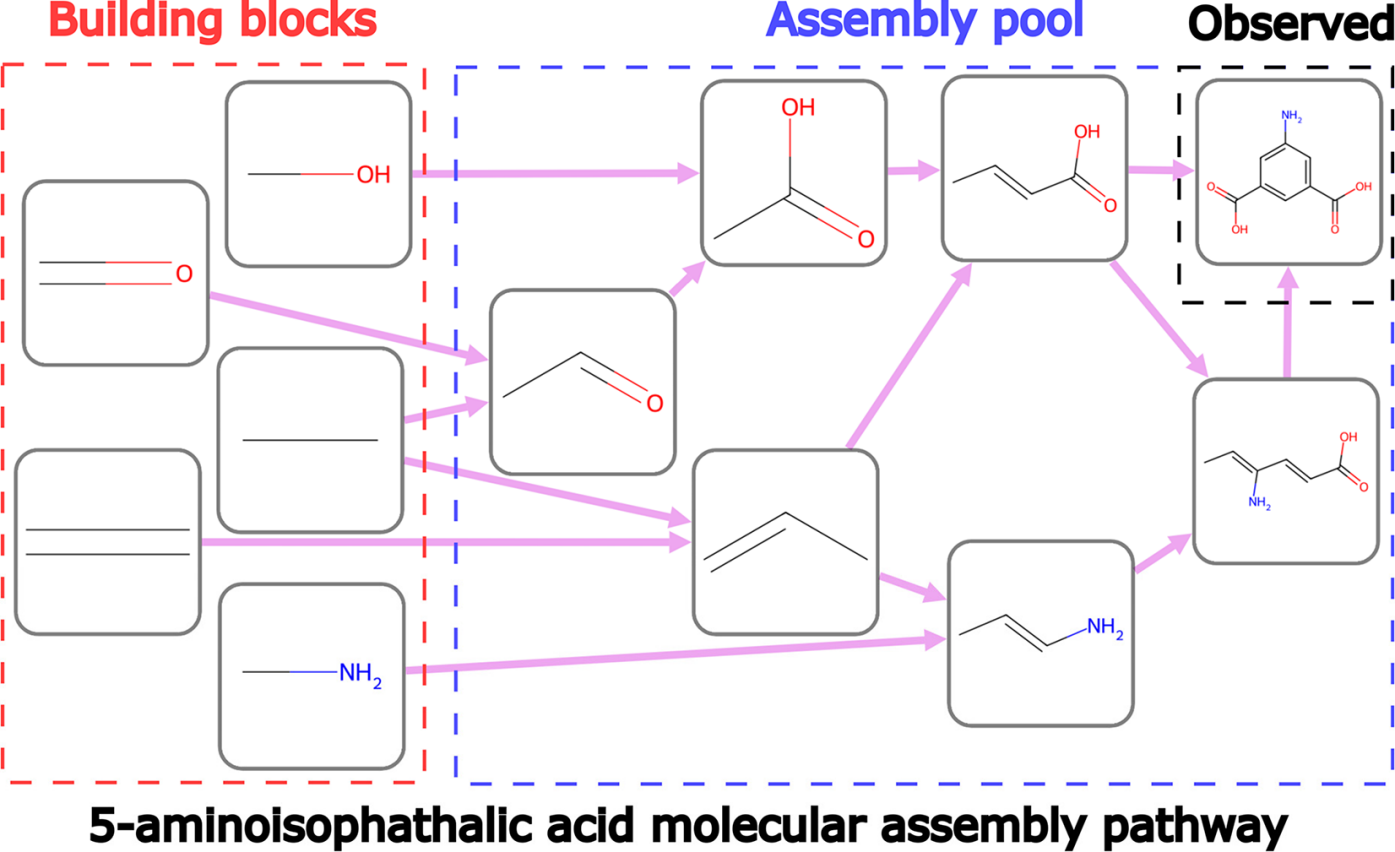
Example molecular assembly pathway for 5-aminoisophathalic acid. The molecular assembly pathway is defined as the fewest number of recursively constructed bond-making operations necessary to produce the given molecular structure. The number of steps on the pathway defines the molecular assembly index (MA). For this example, MA = 7 as seven joining operations are required to assemble the objects in the assembly pool. This pathway was generated using http://www.molecular-assembly.com/query/.

During the 18th century, the Celsius and Fahrenheit scales were developed. These predated the theory of thermodynamics, defining temperature relative to everyday experience, using the melting and boiling of water as reference points. This is not unlike the current state in measuring molecular complexity, which is more heuristic than objective and universal. In the 19th century, William Thomson, better known as Lord Kelvin, also working at the University of Glasgow, used principles from the then-new theory of thermodynamics to define the concept of absolute temperature, with a zero point of no heat, allowing the first absolute scale for temperature. Now, in the recently developed assembly theory, molecular complexity is quantified by the MA index (Figure 1). MA is central to new methods for life detection^4,5^ and a corresponding theory that aims to quantify the complexity of configurations of matter produced by evolutionary processes.^6,7^ Assembly theory provides a neat and straightforward model for the combinatorial generation of innovation in a world of recursive recombining objects, such as that of chemistry. It can be used to study directed vs undirected processes in the creation of molecules, with potential applications in drug discovery, the origin of life, and artificial life. MA is the only molecular complexity measure that is measurable, whereas other molecular complexity measures, including topological, substructure, and graph-theoretic approaches, require prior structural elucidation.^2^ Among these, physicochemical descriptors such as sp^3^-hybridized carbons, the fraction of chiral centers, and their combination^8^ may allow the distinguishing of some natural products, but these also share a challenge in that they lack a strong theoretical motivation for why they should characterize biologically produced complexity. Moreover, there is no absolute scale, and these descriptors are not readily inferred from measurement, meaning they cannot be applied to uncharacterized molecules.

## Anchoring molecular assembly theory in experiments

This work includes a modeling effort with computational results for 10,000 molecules, where the number of peaks in the IR spectra and the types of carbon atoms in the NMR spectra are both shown to correlate well with computed MA. The experimental samples included 99 (IR) and 101 (NMR) compounds for validation. A recursive algorithm using MS/MS fragmentation data to construct a hierarchy of molecular fragments was also shown to estimate the MA with remarkable accuracy, with experimental results confirmed for 101 compounds.

**Figure 2:**
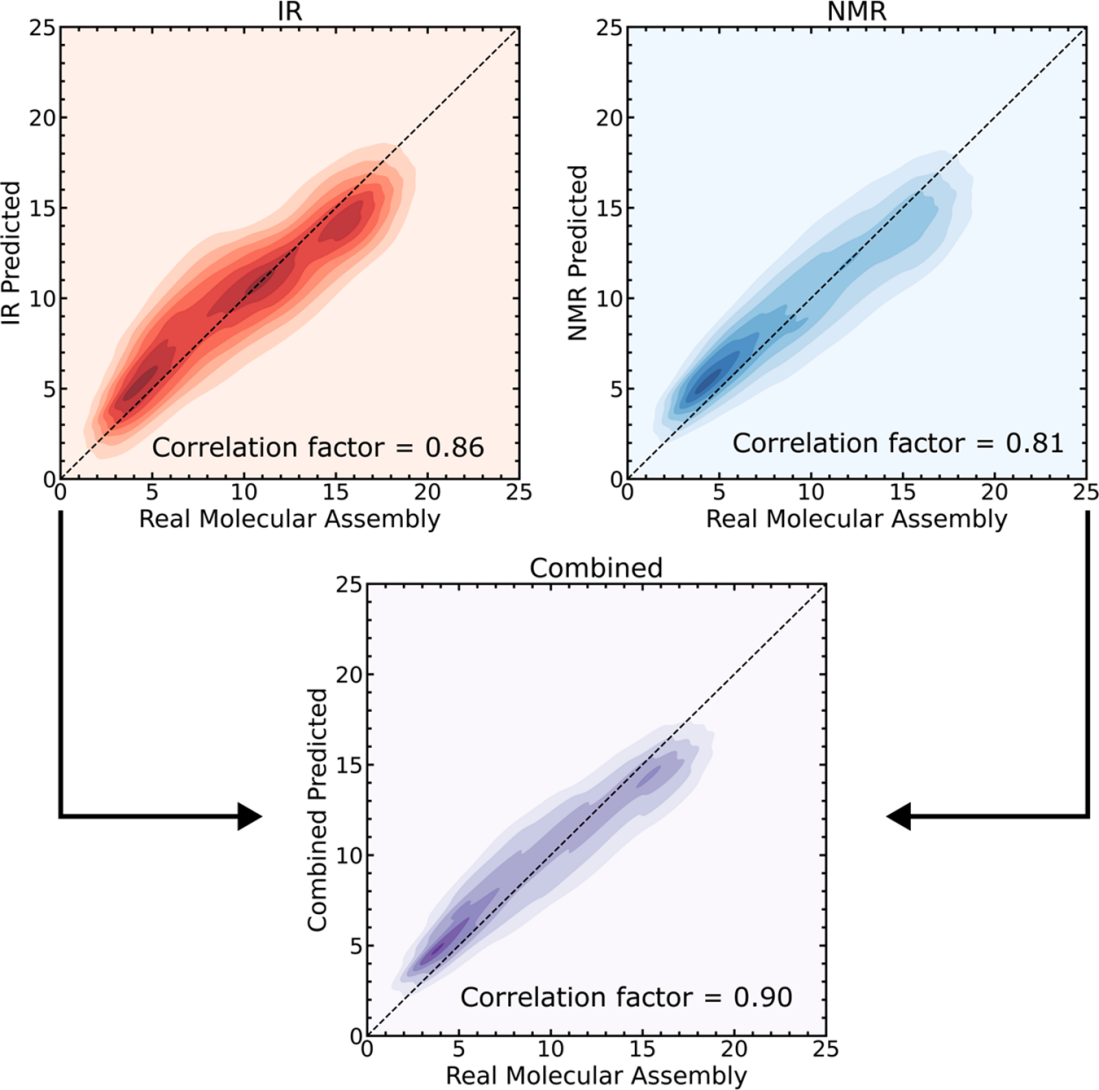
Cronin et al. applied their algorithm at scale to predict MA, implementing a multimodal suite of techniques to demonstrate improved molecular assembly index prediction compared to individual techniques. Reproduced using the data available in Cronin et al.^4^

Furthermore, the authors present a method for rapidly determining MA by combining the three different types of spectroscopic measurements (Figure 2). The authors use 5-aminoisophathalic acid as an example; its MA pathway has seven steps (Figure 1) and includes molecular fragments that combine to form substructures recursively. A central thesis is that MA is determined via the topological encodings in the bonds that can be probed in IR and NMR. Both IR and NMR will agnostically indicate the complexity of a molecule defined by MA since assembly theory states that MA utilizes unique irreducible motifs to construct the molecule that are indicated by the observed spectral features.

Cronin and co-workers evaluated several potential algorithms to determine the MA from spectra. In the case of IR, the number of nonlocal vibrational modes encoded in the fingerprint region (400–1500 cm^–1^) strongly correlates with MA. Using density functional-based tight binding models, a theoretical approach to quickly establish estimates of chemical structures, the authors calculated six strong peaks in the fingerprint region, which is close to the exact MA (MA = 7) for the molecule.

Meanwhile, MA from NMR can be successfully obtained via a weighted sum of the number of carbon resonances sorted by the number of attached hydrogens. The fewer hydrogens attached to the carbon, the higher its weight for MA prediction. In prior work, sp^3^-hybridized carbon counting has been used to infer complexity,^2,8^ but here, the authors show how higher-order carbons can encode complexity: the more hydrogens attached to the carbon, the less localized information it stores and hence the less it contributes to the molecular assembly.

## An agnostic tool to aid in the hunt for biosignatures

Looking for biosignatures on other planetary bodies is an open challenge. Ideally, one could measure a mix of unknown molecules and produce compelling evidence that it was produced by life. This paper takes a step toward this big ask by enabling such predictions and testing them with empirical measurements.

Current complexity measures require structural elucidation, and in biosignature science, we nearly universally have been constrained to look for molecules associated with life on earth. However, MA requires no prior assumption that complex alien chemistry is anything like what evolved on earth. Instead, it provides an objective scale, ranking molecules based on their assembly index. The conjecture of the theory is that the abiotic probability of a molecule’s construction is exponentially less likely with each additional step in its minimal pathway such that high MA molecules will never be found in detectable abundance outside of bioprocesses. Thus, if high assembly index molecules above a threshold for life^6^ are detected, then they should be indicative of life.

Now validated, the approach can be implemented without needing complete structural elucidation; this enables using MA as a standard metric to explore complex chemical spaces and identify signatures of evolutionary processes, even in systems where we do not know the exact chemical composition. Applications include identifying the complexity of uncharacterized or uncharacterizable metabolites, such as in underground metabolism,^9^ unculturable organisms, or the biogeochemical environment. More importantly, it opens previously impossible possibilities such as detecting the emergence of life in complicated chemical mixtures or alien life in other worlds without *a priori* anthropomorphic bias.

When Thompson devised his objective scale for temperature, the new science of thermometry did not end; instead, that is when a new field really began. It often takes decades for mature science to develop and for us within the scientific community to collectively work to understand the ideas we generate. However, grounding those ideas in measurement is a critical first step to unlocking a new frontier. It will be exciting to see what transformations to our understanding of the complexity of the chemical universe will unfold in the coming decades as the metrology of complexity matures, with standard metrics for understanding the world of complex molecular forms unfolding around us.
